# Three‐dimensional gamma criterion for patient‐specific quality assurance of spot scanning proton beams

**DOI:** 10.1120/jacmp.v16i5.5683

**Published:** 2015-09-08

**Authors:** Chang Chang, Kendra L. Poole, Anthony V. Teran, Scott Luckman, Dennis Mah

**Affiliations:** ^1^ ProCure Proton Therapy Center Somerset NJ 08873 USA

**Keywords:** proton therapy, pencil beam scanning, 3D gamma, patient‐specific QA

## Abstract

The purpose of this study was to evaluate the effectiveness of full three‐dimensional (3D) gamma algorithm for spot scanning proton fields, also referred to as pencil beam scanning (PBS) fields. The difference between the full 3D gamma algorithm and a simplified two‐dimensional (2D) version was presented. Both 3D and 2D gamma algorithms are used for dose evaluations of clinical proton PBS fields. The 3D gamma algorithm was implemented in an in‐house software program without resorting to 2D interpolations perpendicular to the proton beams at the depths of measurement. Comparison between calculated and measured dose points was carried out directly using Euclidian distance in 3D space and the dose difference as a fourth dimension. Note that this 3D algorithm faithfully implemented the original concept proposed by Low et al. (1998) who described gamma criterion using 3D Euclidian distance and dose difference. Patient‐specific proton PBS plans are separated into two categories, depending on their optimization method: single‐field optimization (SFO) or multifield optimized (MFO). A total of 195 measurements were performed for 58 SFO proton fields. A MFO proton plan with four fields was also calculated and measured, although not used for treatment. Typically three different depths were selected from each field for measurements. Each measurement was analyzed by both 3D and 2D gamma algorithms. The resultant 3D and 2D gamma passing rates are then compared and analyzed. Comparison between 3D and 2D gamma passing rates of SFO fields showed that 3D algorithm does show higher passing rates than its 2D counterpart toward the distal end, while little difference is observed at depths away from the distal end. Similar phenomenon in the lateral penumbra was well documented in photon radiation therapy, and in fact brought about the concept of gamma criterion. Although 2D gamma algorithm has been shown to suffice in addressing dose comparisons in lateral penumbra for photon intensity‐modulation radiation therapy (IMRT) plans, results here showed that a full 3D algorithm is required for proton dose comparisons due to the existence of Bragg peaks and distal penumbra. A MFO proton plan with four fields was also measured and analyzed. Sharp dose gradients exist in MFO proton fields, both in the middle of the modulation and toward the most distal layers. Decreased 2D gamma passing rates at locations of high dose gradient are again observed as in the SFO fields. Results confirmed that a full 3D algorithm for gamma criterion is needed for proton PBS plan's dose comparisons. The 3D gamma algorithm is implemented by an in‐house software program. Patient‐specific proton PBS plans are measured and analyzed using both 3D and 2D gamma algorithms. For measurements performed at depths with large dose gradients along the beam direction, gamma comparison passing rates using 2D algorithm is lower than those obtained with the full 3D algorithm.

PACS number: 87.53.Bn, 87.53.Jw, 87.55.de, 87.55.kd, 87.55.ne, 87.55.Qr

## I. INTRODUCTION

Proton therapy has become one of the preferred modes of radiation treatment due to the absence of exit dose.^1^, ^2^, ^3^, ^4^, ^5^ This characteristic makes proton therapy especially attractive for pediatric patients for whom secondary cancer is of concern, and for head and neck retreatments where adjacent critical organs may be already at limit. This physical difference in depth‐dose distributions between proton and photon radiation also presents new challenges, such as range uncertainty and increased dose gradients along the beam direction. The accuracy of proton dose calculation under range uncertainty^6^, ^7^, ^8^ and the robustness of plan quality with increased dose gradients^9^, ^10^, ^11^, ^12^ have both been shown to be important factors in patient treatment. In this study, we report the effect of dose gradients along the beam direction on the patient‐specific quality assurance of dose distribution.

The existence of Bragg peaks and the resulting distal dose falloffs is unique to particle beam radiation therapy.^13^, ^14^ While both X‐ray and proton radiation can be modulated in the lateral direction by beam limiting devices, such as apertures and multileaf collimators (MLCs), protons can be effectively modulated along the beam direction by varying the proton energy. Proton dose distributions are, therefore, distinct in that their distal penumbra can be used for dose conformation. As a result, both lateral and distal dose variations (e.g., lateral and distal penumbrae) need to be considered simultaneously when evaluating doses from proton beams. This is different from photon IMRT fields, where dose gradients exist mostly on lateral planes perpendicular to the beam and consequently 2D interpolation is capable of capturing most of the dose variations at depth with fidelity. Conventional 2D gamma algorithm that relies on 2D interpolation, which is established for photon IMRT dose comparison, may not be sufficient for proton dose evaluation. Here we present a full 3D gamma algorithm and compare the dosimetric agreements between measured and calculated doses for patient‐specific modulated proton beams for 2D and 3D gamma.

The gamma criterion, as originally defined by Low et al.,^(15)^ simultaneously takes into account the 3D Euclidean distance between calculated and measured dose points, as well as distance to agreement to take into account measured and delivered variations in sharp dose gradients. Its implementation in photon IMRT quality assurance (QA), however, has been simplified to rely on 2D interpolation, using 2D Euclidean distance in space and dose difference as parameters for gamma comparison. This practice works for photon fields due to the relatively shallow photon dose gradient along the beam direction at about 0.4% per mm. For protons, however, with a typical distal falloff of Bragg peaks of approximately 8%−10% per mm, the calculated and measured dose distributions at the distal end of Bragg peaks are significantly more sensitive to accuracy of the interpolation and uncertainty in the water‐equivalent thickness of the phantom. Earlier studies of patient‐specific proton modulated beam QA focused on using 2D algorithm to evaluate dose distributions in the middle of the beam's modulation.^16^, ^17^ Here, we have extended the gamma analysis to 3D Euclidean space and dose difference to effectively determine the agreement in dose distributions for proton fields. This new approach is expected to provide improvements especially at locations where large dose gradients are present longitudinally.

Gamma criterion is the standard in determining the agreement between measured and calculated doses for photon IMRT fields.^18^, ^19^, ^20^, ^21^ It is especially useful in evaluating lateral penumbrae where rapid dose falloffs can cause artificially large percentage errors.^(22)^ By simultaneously considering dose and distance, gamma criterion gives dosimetrically relevant evaluation on actual dose distributions. In contrast to photons, which have only lateral beam limiting capability, protons can be modulated in the beam direction with Bragg peaks. Thus, proton 3D dose distribution for a single beam typically has sharper lateral and distal penumbrae. To evaluate these 3D dose distributions, especially the distal penumbra, one would need a 3D criterion that encompasses not only the lateral uncertainties (such as setup errors) but also distal uncertainties along the beam direction (such as energy straggling). Such full 3D gamma criterion is also less prone to artifacts in regions of high dose gradient and renders clinically relevant dose evaluations.

## II. MATERIALS AND METHODS

For each patient‐specific modulated scanning proton beam, calculated 3D dose distributions are compared to measurements made by an ion chamber array at various water‐equivalent depths. No interpolation is needed, since depth along the beam direction is directly incorporated into the 3D gamma evaluation algorithm. For comparison, 2D gamma evaluations are also performed by interpolating the calculated 3D dose distribution to the water‐equivalent depth of measurement, followed by 2D gamma comparisons in the interpolated plane. For each measurement, both 3D and 2D gamma passing rates are recorded and analyzed.

**Figure 1:**
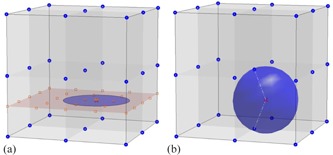
Typical 2D gamma algorithm (a) interpolates the 3D dose grid to a 2D plane at the depth of measurement, and then performs the gamma comparison using 2D Euclidean distance on the interpolation plane and the dose difference. Accuracy of this 2D gamma comparison therefore depends on the veracity of the 2D interpolation. The 3D dose grid (blue ‘o’), the location of ion chamber measurement (red ‘x’), and the interpolated 2D dose grid (red ‘square’) are shown. Full 3D gamma algorithm (b) directly compares the measured dose to the calculated 3D dose grid using 3D Euclidean distance and the dose difference. Calculated 3D dose grid (blue ‘o’) and the point of measurement (red ‘x’) are shown. A 3D sphere of the same radius (i.e., 3 mm here) encompasses points on the 3D dose grid to be considered for gamma comparison with the measurement. This 3D gamma algorithm allows direct comparison of measured to calculated doses without resorting to interpolation.

Figure 1 illustrates the geometry used to formulate the 3D and 2D gamma algorithms described below. Conventional 2D implementation of the gamma criterion requires first an interpolation of the calculated 3D dose to the 2D plane at the depth of measurement. The gamma comparison is then performed between the measured dose values from the ion chamber array and the calculated 2D dose interpolation. As seen in Fig. 1(a), the 3D lattice (○) represents the calculated dose grid, and the 2D plane (red) represents the plane of measurement whose depth corresponds to the water‐equivalent thickness of the phantom used for actual measurement. The size of the 2D interpolation grid is usually chosen to be equal to or finer than the original 3D grid, shown in Fig. 1(a) as 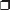. A disk of radius d is then drawn around each point of measurement (×) and gamma comparison is then performed between the measured data point and all 2D interpolated grid points enclosed within the disk, as seen in Fig. 1(a). Note that d is the agreement distance in the gamma criterion, set at 3 mm for all cases described in this article. In practice, only those 2D interpolated grid points enclosed by the disk are included for comparison because all other points render gamma values greater than 1 with distances to the measurement point further than d.

Figure 1(b) depicts the geometry for the 3D gamma algorithm implemented by the in‐house program. No interpolation is needed. Instead a 3D sphere of radius d is drawn centered at each of the measured data point (× in Fig. 1(b)). Note again that d is the same agreement distance in the gamma criterion as seen in Fig. 1(a), and any calculated dose grid points located beyond the sphere would certainly generate a gamma value greater than 1. Gamma criterion is then evaluated for each point of measurement, against all calculated dose points enclosed by the sphere. Since no interpolation is needed, this 3D gamma method is capable of capturing the rapid dose falloff along the beam direction as well as laterally, without subjecting to the errors associated with interpolating to the exact water‐equivalent location of the beam.

All modulated scanning proton beams are calculated using commercially available treatment planning systems (i.e., CMS XiO 5.00.01 (IMPAC Medical Systems, Elekta, Stockholm, Sweden) and RayStation 4.0.3 (RaySearch Laboratories, Stockholm, Sweden)). The XiO system was originally used for PBS planning, but was later replaced by RayStation in our clinic. All plan doses are calculated on 3 mm dose grid, equal in X, Y, and Z directions. Doses are exported in standard Digital Imaging and Communications in Medicine (DICOM) format for comparison with measurements. A commercially available ion chamber array (MatriXX PT, IBA Dosimetry, Belgium) is used for all measurements. The active area of measurement is 24.4 cm by 24.4 cm with 1020 ion chambers in a 32 by 32 grid. Chamber spacing is approximately 7.6 mm by 7.6 mm from center to center, and the saved measurements are interpolated by default to 1 mm by 1 mm resolution. Equivalent water depth of the phantom used for measurement is achieved by placing Plastic Water, with a water‐equivalent thickness of 0.97, (CIRS Inc., Norfolk, VA) in front of the MatriXX. The gamma criterion is applied to each of the measured data points against the calculated dose distribution.

Dose distributions for each of the proton fields are calculated separately onto a virtual 40 cm×40 cm×40 cm water tank to allow for individual field by field comparison with the measurements. For each of the proton fields, 2D dose distributions are measured typically at three different depths. One of the measurements is always made at the middle of the modulation, with at least two other measurements: one upstream and the other downstream of the beam path. A standard absolute dose output measurement is performed prior to each measurement session in order to calibrate the day‐to‐day machine variation. All gamma evaluations, both 2D and 3D, are done with 3%/3 mm criteria with a 10% threshold and Van Dyk normalization.^(18)^ Point of normalization is set at the middle of the field for single‐field optimized (SFO) beams.^(23)^ For multifield optimized (MFO) beams,^(24)^ the normalization point is selected from regions of low dose gradient.

**Figure 2:**
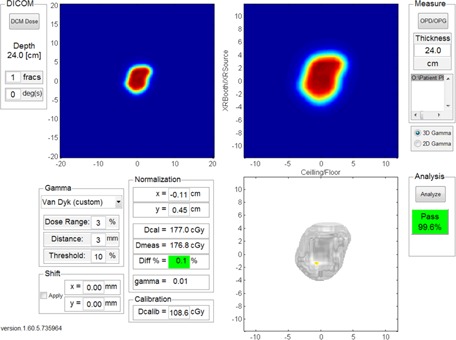
Interface to the in‐house program for 3D gamma criterion comparison.

Figure 2 shows the graphical user interface of the in‐house 3D gamma software. The upper left panel shows the calculated dose imported directly from a standard DICOM dose file. Measured data from the ion chamber array are loaded into the upper right panel. Parameters for gamma criterion, such as agreement distance, dose difference percentage, threshold value, normalization (Van Dyk), and lateral shifts, are all set in panels on the lower left corner. Gamma values are calculated for each measurement point and displayed in the lower right‐hand panel. Percentage of measurement points with gamma value less than or equal to 1 (i.e., the gamma passing rate) is calculated. Currently in our clinic, a gamma passing rate greater than 95% is required for treatment. Both 3D and 2D gamma algorithms are implemented in this in‐house program; and both 3D and 2D gamma passing rates are reported.

## III. RESULTS

For each measurement, both 3D and 2D algorithms are applied to obtain the respective 3D and 2D gamma passing rates. To examine the effectiveness of 3D versus 2D gamma algorithms, these gamma passing rates are plotted as a function of distance from the distal end. Distance from the distal end (i.e., the x‐axis) is defined as the difference between the water‐equivalent depth of the measurement and the range of the beam's most distal layer. As an example, for measurements done at a water‐equivalent depth of $20g/{\text{cm}}^{2}$ for a proton field with a most distal layer of $24.5\;g/{\text{cm}}^{2}$, the distance from the distal end is $-4.5\;g/{\text{cm}}^{2}$. Note that this distance is zero when the measurement is conducted at a depth equal to the range of the beam's most distal layer. Both 3D and 2D gamma passing rates are fitted to the equation

(1) $$R=P(1-e^{\beta (x-c)})$$

where *R* is the measured 3D/2D gamma passing rates; *x* equals to depth subtracted by range of the most distal layer; the least squares fitting parameter *P* is the nominal passing rate for measurements performed away from the distal end; *β* is a measure on the rate of decrease in passing rates as measurements approach the distal end of the beam; and *c* is the offset along the beam direction.

**Figure 3:**
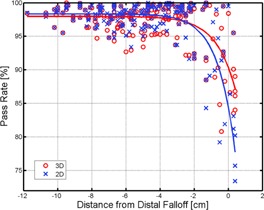
Gamma dose comparison for SFO proton fields. Proton beams from patient‐specific SFO plans are measured. Gamma passing rates, both 3D (red ‘o’) and 2D (blue ‘x’), are recorded and plotted against the distance to the most distal layer (i.e., measurement depth subtracted by the range of the beam's deepest layer). Least square fits to the 3D (red solid line) and 2D (blue solid line) data are also shown.

Figure 3 shows results of 3D versus 2D passing rates plotted as a function of distance from the distal end for 58 separate SFO fields. All fields were from pelvis patients including prostate, GYN, and sarcomas. Data points showing 3D passing rates are designated by red ‘○’ and 2D passing rates by blue ‘×’. Curves fitted to Eq. (1) using 3D and 2D data are also shown as red and blue solid lines, respectively. In general, no significant difference is observed between 3D and 2D gamma passing rates when the measurement is conducted away from the distal end. The 3D and 2D passing rates only become visibly different as the measurement plane approaches the distal end, and the fitted curve did not diverge until it is within $2g/{\text{cm}}^{2}$ of the most distal layer. The nominal passing rate using 3D algorithm is $P_{3D}=98.0\%$, which is indistinguishable from the $P_{2D}$ value of 98.3%. The rate of decrease of the 2D gamma passing rate β_2D_ has a value of 0.98, which is larger than its 3D counterpart $\beta_{3D}=0.87$, indicating a more rapid decrease in 2D passing rates toward the distal end.

**Figure 4:**
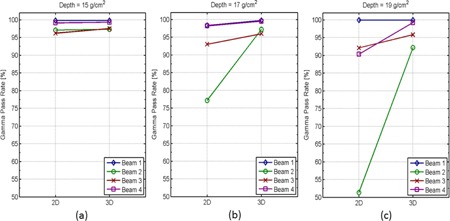
Gamma dose comparison for MFO proton fields. Dose distributions from four fields of a MFO proton plan are measured and compared to their respective calculated doses using both 3D and 2D gamma algorithms. Gamma passing rates obtained from 3D and 2D algorithms are plotted side by side for each field at three different depths: (a) $15g/{\text{cm}}^{2}$, (b) $17g/{\text{cm}}^{2}$, and (c) $18g/{\text{cm}}^{2}$.

Figure 4 shows the gamma dose comparisons of a MFO proton plan with four fields. Ranges of the most distal layers are between 27.9 to $28.3\;g/{\text{cm}}^{2}$, and the most proximal layers between 11.0 to $10.8\;g/{\text{cm}}^{2}$. Measurements are therefore performed at 15, 17, and $19g/{\text{cm}}^{2}$. Essentially no difference (<1.3%) is observed between 3D and 2D gamma passing rates at $15g/{\text{cm}}^{2}$ depth. At $17g/{\text{cm}}^{2}$ depth, Beam 2's 2D gamma passing rate drops to less than 80%, while its 3D gamma passing rate remains at above 95%. For the other three beams at this depth, 3D gamma passing rates are all slightly higher than their respective 2D counterparts, with differences all less than 3.1%. At $19g/{\text{cm}}^{2}$ depth, the difference in 3D and 2D gamma passing rates increased to 40.9% (92.2% for 3D gamma vs. 51.3% for 2D gamma) for Beam 2, 8.9% (99.2% for 3D gamma vs. 90.2% for 2D gamma) for Beam 4, and 3.7% (95.8% for 3D gamma vs. 92.1% for 2D gamma) for Beam 3. Beam 1, however, has its 3D and 2D gamma passing rates equal at 99.8% for $19g/{\text{cm}}^{2}$ depth.

Closer inspection of individual beam's dose distribution reveals that although Beam 2's most distal layer is at $27.9\;g/{\text{cm}}^{2}$, a large portion of its dose distribution along the beam direction actually falls off sharply around $17g/{\text{cm}}^{2}$ at approximately 7.6% per mm, which results in the decrease in its 2D gamma passing rates beyond $17g/{\text{cm}}^{2}$ depth. Beam 1, on the other hand, does not have such dose falloff until depth close to its distal edge of $28.3\;g/{\text{cm}}^{2}$. Dose Distributions for Beams 3 and 4 have more complex patterns in dose gradients along the beam direction and their dose distributions do not fall off until beyond $19g/{\text{cm}}^{2}$ depth; and intermediate passing rate differences between 3D and 2D are, therefore, observed for these two beams. These results show that, for MFO fields, large dose gradients along beam direction can exist in the middle of beam modulation and cause 2D gamma algorithm to render falsely lower passing rates than those obtained from the full 3D algorithm.

## IV. DISCUSSION

Results using 2D gamma algorithm, which requires an intermediate step of interpolation, are prone to errors especially at the distal ends of the proton field where large dose gradients exist in the beam direction. Away from those depths where large dose gradients exist along the beam direction, 2D gamma algorithm is observed to work equally well as its full 3D counterpart. This is shown in Fig. 3 where 2D gamma algorithm renders accurate results for proton SFO fields at depths away from the distal end (i.e., where dose gradients vary minimally along the beam direction). For exactly the same reason, 2D gamma algorithm also yields accurate results for photon IMRT fields with gradual dose gradients along the beam. Nevertheless, while this 2D gamma algorithm has indeed been successful in evaluating photon IMRT dose distributions, its use in proton is limited, especially in the plans where there are distal penumbrae and large dose gradients along the beam direction

Figure 3 demonstrates that resorting to 2D interpolation, as in the 2D gamma algorithm, renders larger errors in dose comparison as the measurement depth approaches the distal end. This reduction in 2D passing rate is attributed to the SFO field's sharp dose gradients in its distal penumbra. Prior developments in gamma calculation algorithm which employed 2D interpolation, while sufficient for photon IMRT whose dose variation along the beam direction is minimal, is ineffective for proton beams where dose variation in the beam direction can be large. A full 3D gamma algorithm is needed to account for the high dose gradients in the distal end of the proton fields and should, therefore, be used for the QA of patient‐specific modulated proton fields.

Similar phenomenon exists in photon IMRT's lateral dose falloffs, where large discrepancy between calculated and measured doses can occur due to the high dose gradients perpendicular to the beam direction. Since dose gradients in photon's lateral penumbra can be as large as 10% per millimeter, it is clear that a point‐by‐point comparison between calculated and measured doses can easily exceed the typical 3% criteria due to minute errors from detector misalignment in setup, interpolation, detector and calculation grids mismatch, uncertainty in the consistency of the solid water used for QA, as well as dose‐volume effect of finite detector size. Indeed, the depth‐by‐depth 2D interpolation of the 3D calculated dose along the proton beam direction, and the subsequent 2D comparison to the measured dose when applied to proton field's distal penumbra, is analogous to the point‐by‐point comparison for the photon beam's lateral penumbra. Similar error in dose comparison is expected as predicted previously^25^, ^26^, ^27^, ^28^, ^29^ and, indeed, demonstrated in Fig. 3.

All fields in a MFO proton plan are optimized simultaneously to achieve target coverage and critical organ sparing. Unlike SFO plans, which optimize each field independently and as a result impose more restrictions on spot placement, MFO plans in general can achieve better homogeneity and organ sparing by optimizing all available spots together. This, however, is at the cost of potentially increased dose heterogeneity within each individual field, while the summed dose distribution of all fields is highly homogeneous. As a result, dose distribution within each MFO field is typically more heterogeneous than that in SFO fields. This increase in MFO's dose heterogeneity exists not only laterally in planes perpendicular to the beam, but also along the beam direction. In addition, all these dose gradients, be it perpendicular or parallel to the beam, might further exacerbate when the plan is recalculated in a water tank for QA where tissue heterogeneity of the patient no longer exists in the calculation. Since MFO's steeper dose gradients can exist anywhere along the beam (i.e., not limited to the distal end), we expect 3D gamma algorithm to be necessary for the QA of MFO proton fields regardless of measurement depth's relative location to the distal edge.

## V. CONCLUSIONS

A full 3D gamma algorithm was implemented by an in‐house software program for clinical use. This 3D algorithm enables direct comparison of dose distributions in 3D Euclidean space without interpolation. Patient data were collected and initial comparative analysis demonstrated the need for 3D gamma algorithm for modulated scanning proton beams. By comparing 3D versus 2D passing rates, it was shown that proton field's rapid dose falloffs, in both lateral and distal penumbrae, were successfully taken into account by the 3D gamma algorithm, but not its 2D counterpart. Effectiveness of this 3D gamma algorithm in evaluating proton dose distribution is, therefore, demonstrated. We anticipate the clinical implementation of this 3D gamma algorithm to be potentially useful for MFO proton plans.
